# 521 Assessing the Utility of Visceral Proteins as Markers of Nutrition

**DOI:** 10.1093/jbcr/irae036.156

**Published:** 2024-04-17

**Authors:** Billy Jay Taylor, Sara Calder, Isabella G Shawe, Claudia Islas, Karen J Richey, Kevin N Foster

**Affiliations:** Valleywise Health, Phoenix, AZ; Arizona Burn Center Valleywise Health, Warrenville, IL; Arizona Burn Center Valleywise Health, Cleveland Heights, OH; Arizona Burn Center/Valleywise Health Medical Center, Phoenix, AZ; Arizona Burn Center Valleywise Health, Phoenix, AZ; Valleywise Health, Phoenix, AZ; Arizona Burn Center Valleywise Health, Warrenville, IL; Arizona Burn Center Valleywise Health, Cleveland Heights, OH; Arizona Burn Center/Valleywise Health Medical Center, Phoenix, AZ; Arizona Burn Center Valleywise Health, Phoenix, AZ; Valleywise Health, Phoenix, AZ; Arizona Burn Center Valleywise Health, Warrenville, IL; Arizona Burn Center Valleywise Health, Cleveland Heights, OH; Arizona Burn Center/Valleywise Health Medical Center, Phoenix, AZ; Arizona Burn Center Valleywise Health, Phoenix, AZ; Valleywise Health, Phoenix, AZ; Arizona Burn Center Valleywise Health, Warrenville, IL; Arizona Burn Center Valleywise Health, Cleveland Heights, OH; Arizona Burn Center/Valleywise Health Medical Center, Phoenix, AZ; Arizona Burn Center Valleywise Health, Phoenix, AZ; Valleywise Health, Phoenix, AZ; Arizona Burn Center Valleywise Health, Warrenville, IL; Arizona Burn Center Valleywise Health, Cleveland Heights, OH; Arizona Burn Center/Valleywise Health Medical Center, Phoenix, AZ; Arizona Burn Center Valleywise Health, Phoenix, AZ; Valleywise Health, Phoenix, AZ; Arizona Burn Center Valleywise Health, Warrenville, IL; Arizona Burn Center Valleywise Health, Cleveland Heights, OH; Arizona Burn Center/Valleywise Health Medical Center, Phoenix, AZ; Arizona Burn Center Valleywise Health, Phoenix, AZ

## Abstract

**Introduction:**

Burn injury leads to a profound catabolic state requiring aggressive nutritional support to facilitate healing, homeostasis, rehabilitation and to decrease infections. However, monitoring efficacy of nutritional support is difficult. Following the serum levels of visceral proteins is common practice, but recent studies have questioned the value of these tests in evaluating nutritional status. The purpose of this study was to examine the utility of visceral proteins as a measure of nutritional assessment and intervention.

**Methods:**

This was a retrospective chart review of adult patients admitted over one year who required enteral feeding and had indirect calorimetry testing. Caloric data and associated visceral protein labs (pre-albumin, albumin, transferrin, and CRP) were collected during their enteral feeding duration. Patients were stratified by respiratory quotient (RQ), “Normal RQ” (RQ = 0.85-1.00) and “Low RQ” (RQ > 0.85), and Caloric % weekly intake, adequate intake (>90%) and inadequate intake (< 90%). Differences between groups were analyzed using a T-test or Wilcoxon rank sum test depending on data normality.

**Results:**

A total of 44 patients met inclusion/exclusion criteria. Mean age was 50 years, mean TBSA was 27 %, mean BMI was 30.3. The majority were male 70% (n=31), mean length of stay was 58.5 days, mean ventilator days were 41 days. Mortality was 27% (n=12). There were no significant differences in visceral protein levels between RQ groups. There was no significant difference between % Caloric intake (adequate vs. inadequate) for the first 4 weeks. In the fifth week there were differences in pre-albumin, transferrin, and C-reactive protein levels between groups, with p-values of 0.02, 0.03, and 0.006, respectively. There was no significant difference in albumin levels at week 5. Analysis beyond 5 weeks was not conducted due to insufficient data.

**Conclusions:**

Historically, visceral proteins such as albumin and pre-albumin have been used as nutrition markers. We now better understand that the albumin pool of the human body is comprised of little newly synthesized albumin, and protein intake has little effect on the value itself. Albumin and pre-albumin are negative acute-phase reactants and are greatly affected by the inflammation seen with burn injuries. We found no significant differences between pre-albumin and RQ. Conversely, there was also no significant difference between pre-albumin and those who were able to meet greater than 90% of their nutrition needs, and those who met less than 90% needs during weeks 1-4 of testing. There was a significant relation between all visceral proteins and caloric intake during week 5. This is likely explained by improved levels of CRP and overall decrease in inflammation.

**Applicability of Research to Practice:**

Our results support that visceral proteins are not good indicators of nutrition status in burn patients, while in a state of inflammation.

